# Successful Leadless Pacemaker Implantation Despite SVC Obstruction

**DOI:** 10.1016/j.jaccas.2025.103364

**Published:** 2025-04-23

**Authors:** Naveen Vuppuluri, Tarek Makki, Firas Zahwe

**Affiliations:** Department of Cardiovascular Medicine, Henry Ford Warren Hospital, Warren, Michigan, USA

**Keywords:** biotechnology, bradycardia, cardiac pacemaker, cardiomyopathy, vascular disease

## Abstract

**Background:**

A 69-year-old male underwent implantation of a leadless pacemaker after a failed attempt to upgrade his single lead ICD in the setting of SVC obstruction.

**Case Summary:**

A 69-year-old male with ischemic cardiomyopathy status post single chamber ICD presented with fatigue. Device interrogation revealed increased RV pacing. Device upgrade was attempted; however, he was found to have a SVC obstruction. A leadless pacemaker was subsequently placed in the right atrium via femoral approach.

**Discussion:**

Permanent leadless pacemakers provide a safe, less invasive alternative for patients with conditions that would have previously required complex interventions. Our case demonstrates the use of a leadless pacemaker in a patient with SVC obstruction and ESRD, offering lower infection risk and benefit of retrievability.

## Graphical Abstract

**Figure:**
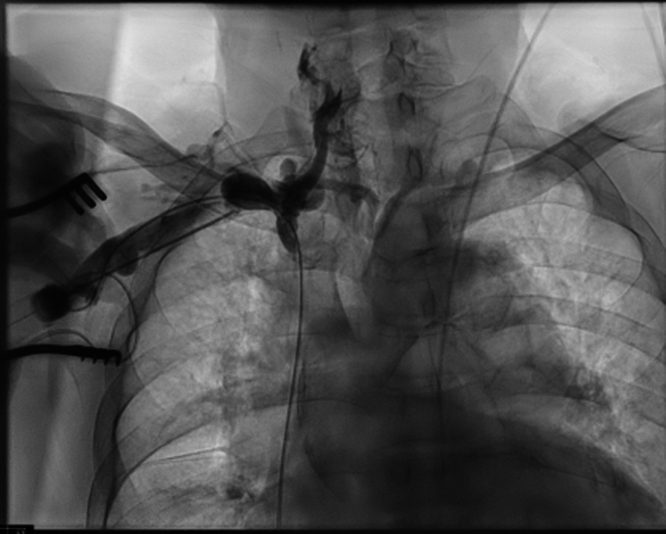

## History of presentation

The patient is a 69-year-old male with ischemic cardiomyopathy status post single-chamber implantable cardioverter-defibrillator (ICD) in 2022 for primary prevention. His case had been lost to follow-up after ICD implantation but was seen again for complaints of fatigue in 2024. An in-office electrocardiogram revealed sinus bradycardia. An additional device interrogation revealed increased right ventricular pacing at 67.42%. Because of the bradycardia and high right ventricular pacing, a device upgrade to a dual-chamber ICD was recommended. The original ICD in 2022 was placed via the right subclavian vein using a nonthoracotomy approach with initial device programming VVI at a rate of 40 beats/min with a ventricular fibrillation zone above 200 beats/min.

**Take-Home Messages**

• Using novel cardiac device therapies as safe and effective alternatives for patients requiring ICD system upgrades is important.
• Staying up-to-date with technology is essential for clinicians to enhance patient care and offer unique solutions.

## Past medical history

Past medical history was significant for ischemic cardiomyopathy status post single-chamber ICD in 2022 for primary prevention, heart failure with reduced ejection fraction of 15%, and end-stage renal disease on hemodialysis.

## Investigations

**Figure 1:**
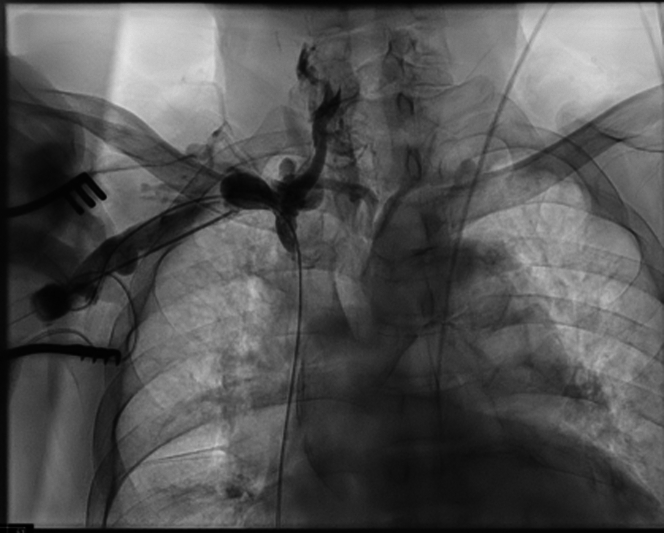
A Venogram Through the Cook Needle Showed Complete Obstruction of the Superior Vena Cava

The patient was subsequently brought to the electrophysiology lab and an upgrade to a dual-chamber ICD was attempted using a right subclavian nonthoracotomy approach and modified Seldinger technique. Attempts at subclavian access were made using fluoroscopy and a Cook needle. A J-wire, angled glide, and Micropuncture wire could not be advanced through the superior vena cava. A venogram through the Cook needle showed complete obstruction of the superior vena cava (Figure 1). At this time, the procedure was aborted and the decision was made to place an AVEIR AR Leadless Pacemaker in the right atrium via a femoral approach.

## Management

**Figure 2:**
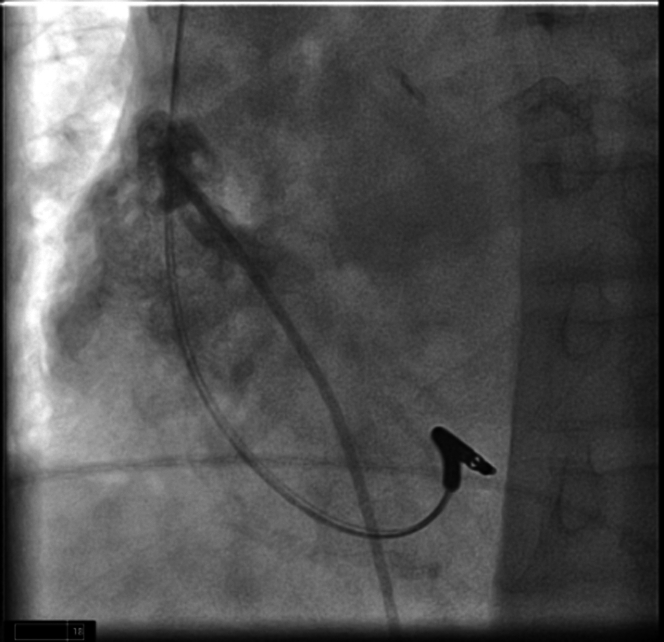
Pigtail Contrast Images Pigtail contrast images were taken in left anterior oblique at 30 degrees to identify the target location for the AVEIR AR Leadless Pacemaker which was at the base of right atrial appendage.

**Figure 3:**
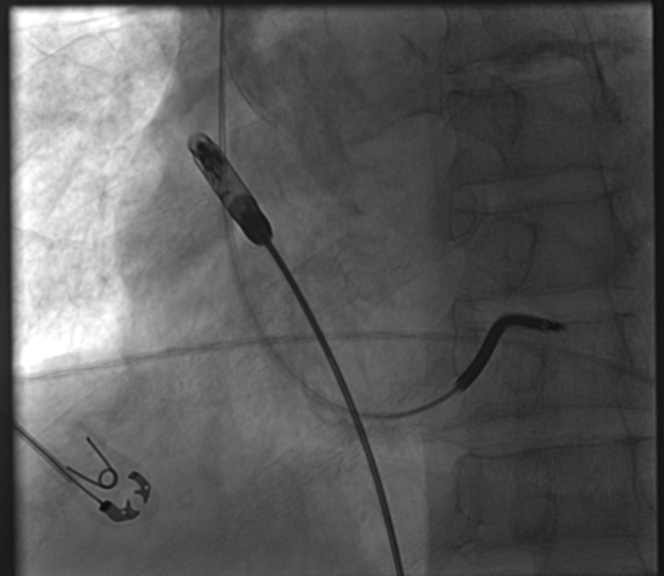
Electrical Mapping The AVEIR AR device was exposed against the lateral base of the right atrial appendage in the left anterior oblique region and electrical mapping was initiated.

**Figure 4:**
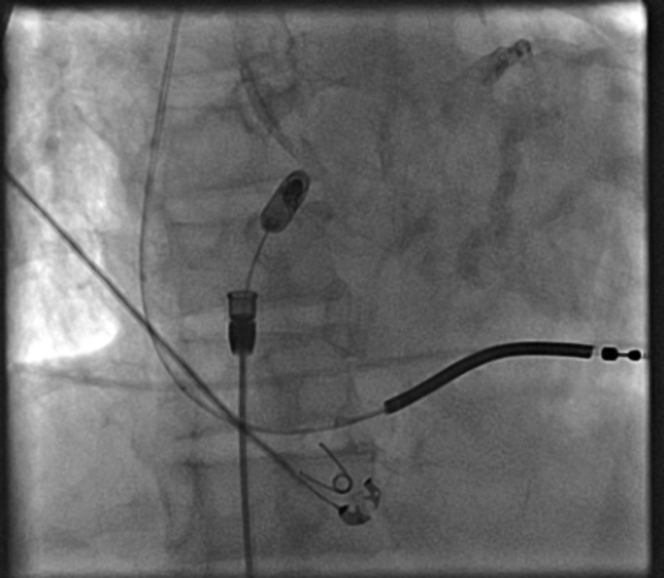
Tension Test A “tension test” was performed in the right anterior oblique region and recorded on fluoroscopy.

The patient was brought back to the electrophysiology lab in a fasting nonsedated state and placed on the table in supine position. Sedation was achieved with Versed and fentanyl. After local infiltration with 1% lidocaine, a single right femoral venous access was obtained using ultrasound. An 8-F sheath was placed in the right femoral vein. This sheath was then upsized to a 27-F sheath over a super stiff Amplatz wire. Pigtail contrast images were obtained in right anterior oblique (RAO) and left anterior oblique (LAO) at 30° to identify the target location which was the base of right atrial appendage for the AVEIR AR Leadless Pacemaker (Figure 2). The leadless pacemaker delivery system was prepped and advanced under fluoroscopic guidance up to the right atrial appendage and confirmed by angiogram. The AVEIR AR was exposed in the high inferior vena cava/low atrium initiating communication with the programmer. RAO/LAO contrast images were obtained via delivery catheter confirming location. The AVEIR AR device was exposed against the lateral base of the right atrial appendage and electrical mapping was initiated (Figure 3). After acceptable measurements were obtained, the device was fixed and the delivery catheter was manipulated into tether mode. A “tension test” was performed and recorded on fluoroscopy (Figure 4). Post fixation and tether device measurements were reviewed and confirmed with an additional set following a 5-minute waiting period. The AVEIR AR device was then deployed at the base of the right atrial appendage and final electrical data were obtained. The device was programmed to AAIR 60-100.

## Outcome and follow-up

An ICD interrogation was performed to verify adequate function of the original device in the presence of the AVEIR AR Leadless Pacemaker. The procedure was tolerated and the patient was discharged home in a stable condition.

## Discussion

The advent of permanent leadless pacemaker implantation has greatly expanded the clinician’s ability to offer necessary therapies to their patients. Cases similar to ours would have previously undergone extensive interventions to receive the necessary device upgrade.^1^^,^^2^ These alternative interventions were discussed and included lead extraction followed by stenting of the superior vena cava (SVC) occlusion with re-implantation of the ICD. This option was not pursued because it would require multiple procedures along with the possibility of re-occlusion of the SVC. Furthermore, the patient had a high risk of infection due to having end-stage renal disease on hemodialysis. Proceeding with implantation of an AVEIR AR Leadless Pacemaker was determined to have less infection risk and lower overall complication rate. Using this novel device enables us to bypass many of these interventions and offer a safe and effective alternative. Our case highlights the ability to use permanent leadless atrial pacemakers in the setting of an SVC obstruction that precludes placement of a traditional pacemaker. Furthermore, we show that the permanent leadless atrial pacemaker can function in the setting of an additional pacemaker/ICD system. Additionally, we review the procedural aspects in placing the permanent leadless atrial pacemaker and the multiple safety aspects to ensure a successful procedure while limiting complications. An additional benefit of the AVEIR AR device is that it is retrievable, which makes it an attractive option for younger patients who will likely need multiple devices during their lifetime due to battery depletion.

## Conclusions

This case emphasizes the importance of staying up-to-date with current cardiac device therapy and highlights the clinicians ability to apply “outside the box” thinking in managing complex cardiac patients.

## Funding Support and Author Disclosures

The authors have reported that they have no relationships relevant to the contents of this paper to disclose.
